# Structural Investigations of *N*-carbamoylputrescine Amidohydrolase from *Medicago truncatula*: Insights into the Ultimate Step of Putrescine Biosynthesis in Plants

**DOI:** 10.3389/fpls.2016.00350

**Published:** 2016-03-30

**Authors:** Bartosz Sekula, Milosz Ruszkowski, Maura Malinska, Zbigniew Dauter

**Affiliations:** ^1^Institute of Technical Biochemistry, Faculty of Biotechnology and Food Sciences, Lodz University of TechnologyLodz, Poland; ^2^Synchrotron Radiation Research Section, Macromolecular Crystallography Laboratory, National Cancer Institute, ArgonneIL, USA; ^3^Faculty of Chemistry, University of WarsawWarsaw, Poland

**Keywords:** polyamine synthesis, amidase mechanism, cadaverine, 1, 4-diaminobutane, crystal structure, octamer, carbamoyl hydrolysis, CPA

## Abstract

Putrescine, 1,4-diaminobutane, is an intermediate in the biosynthesis of more complexed polyamines, spermidine and spermine. Unlike other eukaryotes, plants have evolved a multistep pathway for putrescine biosynthesis that utilizes arginine. In the final reaction, *N*-carbamoylputrescine is hydrolyzed to putrescine by *N*-carbamoylputrescine amidohydrolase (CPA, EC 3.5.1.53). During the hydrolysis, consecutive nucleophilic attacks on the substrate by Cys158 and water lead to formation of putrescine and two by-products, ammonia and carbon dioxide. CPA from the model legume plant, *Medicago truncatula* (*Mt*CPA), was investigated in this work. Four crystal structures were determined: the wild-type *Mt*CPA in complex with the reaction intermediate, *N*-(dihydroxymethyl)putrescine as well as with cadaverine, which is a longer analog of putrescine; and also structures of *Mt*CPA-C158S mutant unliganded and with putrescine. *Mt*CPA assembles into octamers, which resemble an incomplete left-handed helical twist. The active site of *Mt*CPA is funnel-like shaped, and its entrance is walled with a contribution of the neighboring protein subunits. Deep inside the catalytic cavity, Glu48, Lys121, and Cys158 form the catalytic triad. In this studies, we have highlighted the key residues, highly conserved among the plant kingdom, responsible for the activity and selectivity of *Mt*CPA toward *N*-carbamoylputrescine. Moreover, since, according to previous reports, a close *Mt*CPA relative from *Arabidopsis thaliana*, along with several other nitrilase-like proteins, are subjected to allosteric regulation by substrates, we have used the structural information to indicate a putative secondary binding site. Based on the docking experiment, we postulate that this site is adjacent to the entrance to the catalytic pocket.

## Introduction

Polyamines (PAs) are small, aliphatic, polycationic compounds that bear at least two amino groups. Although PAs are found in all domains of life, the most abundant PA in nature is 1,4-diaminobutane, commonly named putrescine (PUT), which, together with 1,5-diaminopentane, cadaverine (CAD), owes its name to the noxious odor of putrefying cadavers. More complexed PAs include, but are not restricted to, triamine spermidine (SPD), tetraamine spermine (SPM) and thermospermine. It has been shown that PAs are some of the key players during plant growth and development, as well as response to both abiotic and biotic stresses (Do et al., 2014; Jimenez-Bremont et al., 2014; Minocha et al., 2014; Tiburcio et al., 2014; Liu et al., 2015). To list only a few examples, exogenous application of PUT can enhance thermotolerance of wheat (Kumar et al., 2014) and alleviate salt stress in apple callus (Liu et al., 2006). Also, supplementation with SPD mitigated salt stress symptoms in tomato and sorghum (Zhang et al., 2015; Yin et al., 2016). Application of exogenous PAs was shown to be not the only way to improve stress tolerance by increasing PA levels. Overexpression of genes involved in PA biosynthesis results in accumulation of PAs and, therefore, greater stress tolerance (Roy and Wu, 2001; Capell et al., 2004; Kasukabe et al., 2004; Alcazar et al., 2010; Wang et al., 2011). PAs are also related to nodulation, i.e., the symbiotic interaction between a plant hosts from legume (*Fabaceae*) family and nitrogen-fixing bacteria. *Medicago truncatula (Mt)*, which is the source organism of the enzyme studied in this work, is a model legume plant. Legumes form special organs, root nodules, which are dwelled by nitrogen-fixing bacteria from *Rhizobium* genus. This extraordinary plant–microbe symbiosis allows legume plants to overcome limited nitrogen availability because rhizobia utilize atmospheric N_2_ and convert it into ammonia, which plants are able to assimilate. It has been demonstrated that the concentration of PAs affects both number and biomass of root nodules (Vassileva and Ignatov, 1999; Terakado et al., 2006). Moreover, PA levels in the nodule tissue are elevated by a factor of 5–10, when compared to the rest of the plant organs (Fujihara et al., 1994; Efrose et al., 2008).

For years, the molecular function of PAs was attributed only to their cationic character, that is, affinity to interact with negatively charged biomolecules, such as nucleic acids and proteins, and modulate transcription and translation (Bachrach, 2010; Gill and Tuteja, 2010; Igarashi and Kashiwagi, 2010; Tiburcio et al., 2014). Meanwhile, PAs can also regulate membrane transport (Pottosin and Shabala, 2014; Pottosin et al., 2014a) or stimulate antioxidant systems and quenching of reactive oxygen species (Shi et al., 2010; Xu et al., 2011; Radhakrishnan and Lee, 2013; Shu et al., 2013; Kamiab et al., 2014; Mostofa et al., 2014; Pottosin et al., 2014b).

**FIGURE 1:**
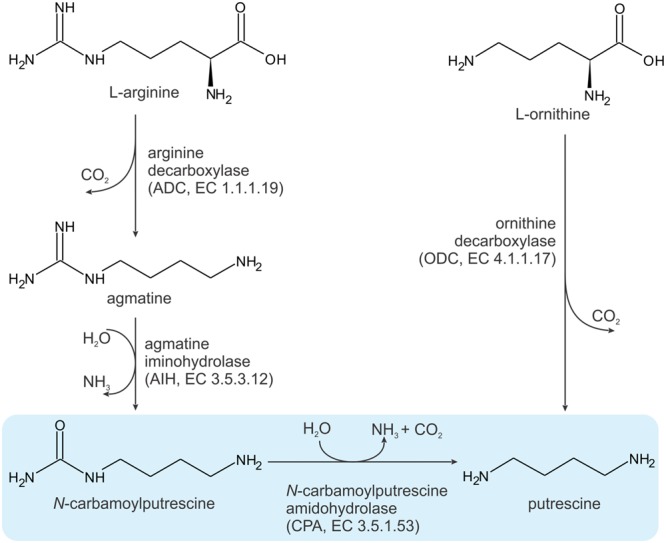
**Two pathways for putrescine biosynthesis.** The reaction catalyzed by *Mt*CPA is highlighted in blue.

In plant cells, the homeostasis of PAs is attained by regulation of their biosynthesis, catabolism, conjugation and transport (Liu et al., 2015). The elementary PA that plants biosynthesize is PUT. Synthesis of SPD and SPM involves enzymatic conjunction of PUT with aminopropyl groups from decarboxylated *S*-adenosylmethionine. There are two routes leading to PUT synthesis (**Figure 1**) (Martin-Tanguy, 2001). One originates from ornithine, which, in a single reaction, catalyzed by ornithine decarboxylase (ODC, EC 4.1.1.17), is converted into PUT. In the other pathway, arginine is the starting molecule, and some plants, such as *Arabidopsis thanliana (At)*, rely solely on this method (Hanfrey et al., 2001). The arginine route involves three enzymatic steps: (i) arginine decarboxylation to agmatine by arginine decarboxylase (ADC, EC 4.1.1.19); (ii) deimination of agmatine by agmatine iminohydrolase (AIH, EC 3.5.3.12) and (iii) hydrolysis of *N*-carbamoylputrescine (NCP) to putrescine by *N*-carbamoylputrescine amidohydrolase (CPA, EC 3.5.1.53), with the release of carbon dioxide and ammonia. The latter enzyme, CPA from *Medicago truncatula* (*Mt*CPA), is the subject of the studies presented here.

Plants are the only eukaryotes able to produce PUT from arginine, which makes enzymes involved in PUT biosynthesis good targets for herbicide design. It has been shown that the plant pathway originates from bacteria (Illingworth et al., 2003; Fuell et al., 2010). Even though all three enzymes, ADC, AIH and CPA are examples of endosymbiotic horizontal gene transfer, they are encoded by nuclear DNA. It is, however, not surprising, as for instance a big part of cyanobacterial genes has been incorporated into the *A. thaliana* genome (Martin et al., 2002). Only ADC contains chloroplast-targeting signal peptide, whereas AIH and CPA are present in cytoplasm (Illingworth et al., 2003). Consistently, only the ADC sequence is present in many cyanobacteria, meanwhile most cyanobacteria use single agmatine ureohydrolase (EC 3.5.3.11) instead of AIH and CPA (Fuell et al., 2010).

*N*-carbamoylputrescine amidohydrolase enzymes belong to nitrilases, and more precisely, to C-N hydrolases breaking non-peptide bonds using Cys-Glu-Lys catalytic triad (Bork and Koonin, 1994; Pace and Brenner, 2001; Chen et al., 2003). Enzymatic activity of *A. thaliana* CPA (*At*CPA) has been examined by Piotrowski et al. (2003). Using protein overexpressed in *Escherichia coli* cells, authors reported that *At*CPA does not follow Michaelis–Menten kinetics, and its enzymatic activity can be described by Hill equation. The reported Hill coefficient, equal 2.2, indicated positive cooperative binding. The maximal velocity, V_max_ was calculated as 86 nanokatals per mg of protein, and half maximal velocity was achieved at 135 μM of NCP. Based on native gel electrophoresis, authors also show that *At*CPA in solution forms homooctamers (Piotrowski et al., 2003).

Even though the overall mechanism catalyzed by nitrilase enzymes has been established, particular subclasses must have evolved several modifications that allow for binding chemically different substrates. So far, there have been no structural information on any CPA enzyme from whichever domain of life. Therefore, it has been unknown how CPAs catalyze the enzymatic reaction, and which amino acid residues interact with the substrate and/or product. It also has been elusive what is the mechanism of the positive cooperative binging. In this studies, we address those fundamental questions, and using the first crystal structures of a CPA protein, expand the knowledge about these housekeeping enzymes.

## Materials and Methods

### Cloning, Overexpression and Purification of *Mt*CPA

*Mt*CPA protein was produced using a protocol adapted from the reported previously for obtaining δ^1^-pyrroline-5-carboxylate reductase from the same organism, (Ruszkowski et al., 2015). *M. truncatula* leaves were used for isolation of the total RNA with RNeasy Plant Mini Kit (Qiagen). cDNA was obtained with the use of SuperScript II reverse transcriptase (Life Technologies) and oligo dT (15 and 18) primers. PCR with the primers (Forward: TACTTCCAATCCAATGCCATGGCGGAAGACAAGGGTAGAAAAGTA and Reverse: TTATCCACTTCCAATGTTATCACAGAACAGGATTTTTGCCGTCCAAA) and cDNA as a template was performed to obtain the coding sequence of *Mt*CPA open reading frame. The PCR product was incorporated into pMCSG68 vector (Midwest Center for Structural Genomics) by ligase-independent cloning (Kim et al., 2011). The N-terminus of the expressed protein contains His_6_-tag, followed by the TEV protease cleavage site, and non-cleavable Ser-Asn-Ala linker that precedes the genuine sequence of *Mt*CPA.

BL21 Gold *E. coli* competent cells (Agilent Technologies), transformed with the vector, were used for overexpression of the protein. Culture was carried out to the A_600_ of 1.0 at 37°C in LB media with the addition of ampicillin (150 μg/ml). The culture was cooled to 18°C before induction. Then, IPTG (final concentration of 0.5 mM) was added to the culture. The culture was grown for 20 h and then cooled to 4°C. Cells were pelleted by centrifugation at 3500 × *g* for 20 min. After the supernatant removal, cells were resuspended in 35 ml of the binding buffer (50 mM Tris-HCl pH 8.0; 500 mM NaCl; 20 mM imidazole; 1 mM TCEP) and frozen at -80°C. Thawed culture was subjected to sonication to disrupt the cells. Bursts of total 4 min duration, with appropriate intervals for cooling, were applied. Disrupted cells were pelleted by centrifugation at 25 000 × *g* for 30 min at 4°C. The supernatant was used to retrieve the His_6_-tagged *Mt*CPA protein on the column packed with 5 ml of HisTrap HP resin (GE Healthcare), which was connected to the Vacuum Manifold (Promega). After application of the supernatant, column was washed five times with 40 ml of the binding buffer. The elution of *Mt*CPA was performed with 20 ml of elution buffer (50 mM Tris-HCl pH 8.0; 500 mM NaCl; 300 mM imidazole; 1 mM TCEP). His_6_-tagged TEV protease was added to the sample to the final concentration of 0.1 mg/ml to cleave the His_6_-tag. The sample was dialyzed overnight at 4°C against the buffer: 50 mM Tris-HCl pH 8.0; 500 mM NaCl; 1 mM TCEP. HisTrap HP resin was used to remove the cleaved His_6_-tag and the His_6_-tagged TEV protease. Final purification was performed by the size exclusion chromatography on HiLoad Superdex 200 16/60 column (GE Healthcare), equilibrated with the buffer: 50 mM Tris-HCl pH 8.0, 200 mM NaCl and 1 mM TCEP. The column was connected to the AKTA FPLC system (Amersham Biosciences).

*Mt*CPA-C158S mutant was obtained by introduction of the C158S mutation to the vector used for overexpression of wild-type *Mt*CPA, according to the Polymerase Incomplete Primer Extension method (Klock and Lesley, 2009). Primers used for PCR reaction were designed as follows – Forward: GCTATTTCCTGGGATCAGTGGTTTCCGGA and Reverse: ATCCCAGGAAATAGCGACTCCAATTTTCGCATA. The same protocol of overexpression and purification as for *Mt*CPA was used in case of *Mt*CPA-C158S. Sequences of *Mt*CPA and *Mt*CPA-C158S mutant were confirmed by DNA sequencing.

### Crystallization and Data Collection

Both, *Mt*CPA and *Mt*CPA-C158S mutant proteins, were concentrated with Amicon concentrators (Millipore) to 20 mg/ml, as determined by the absorbance measurement at 280 nm, with the extinction coefficient of 38640. Initial crystallization trials were performed by sitting drop vapor diffusion method using the crystallization robot (Mosquito). Conditions were established for *Mt*CPA as the 64^th^ reagent of PEG/Ion Screen HT (Hampton research). Both proteins were then crystallized manually by hanging drop method in the conditions containing 20% PEG3350 and 8% Tacsimate at pH 7.0 (Hampton Research) or 20% PEG3350, 8% Tacsimate at pH 7.0 and 10% glycerol (GOL). Crystals with the best morphology were obtained by streak seeding.

Complexes of *Mt*CPA with *N*-(dihydroxymethyl)putrescine (DHMP) and *Mt*CPA-C158S with PUT were obtained by incubation of the unliganded proteins with PUT (10 mM final concentration) for 15 min. Samples were then centrifuged at 17 000 × *g* before crystallization setup. The complex of *Mt*CPA with CAD was formed using the same protocol, with the final 17 mM concentration of CAD. Crystals were cryoprotected by transfer to the solution containing 20% PEG3350, 8% Tacsimate at pH 7.0 and 25% GOL, supplemented with the appropriate ligand.

The diffraction data were collected at Southeast Regional Collaborative Access Team (SER-CAT) 22-ID beamline at the Advanced Photon Source, Argonne National Laboratory, USA. The diffraction images were processed with *XDS* (Kabsch, 2010); for details see **Table 1**.

**Table 1:** Data-collection and refinement statistics.

Structure:	*Mt*CPA/DHMP	*Mt*CPA/CAD	*Mt*CPA-C158S unliganded	*Mt*CPA-C158S/PUT
**Data collection**				
Wavelength (Å)	1.0000	1.0000	1.0000	1.0000
Temperature (K)	100	100	100	100
Space group	*P*2_1_2_1_2	*P*2_1_2_1_2	*P*2_1_2_1_2	*P*2_1_2_1_2
Unit cell parameters *a, b, c* (Å)	152.1, 211.1, 208.8	152.3, 211.0, 208.8	152.5, 210.8, 208.7	152.2, 211.1, 208.6
Oscillation range (°)	0.5	0.5	0.5	0.5
No. of images	200	200	250	180
Resolution (Å)	40-1.97 (2.10-1.97)	40-2.19 (2.32-2.19)	50-2.39 (2.54-2.39)	40-2.29 (2.42-2.29)
Reflections collected/unique	1932219/462097	1416361/340117	1365784/261464	1115016/294393
Completeness (%)	98.3 (91.2)	99.1 (95.3)	99.3 (97.3)	97.5 (88.0)
Multiplicity	4.2 (4.2)	4.2 (4.1)	5.2 (5.2)	3.8 (3.8)
*R*_merge_(%)	9.8 (74.1)	10.8 (75.4)	13.9 (73.8)	11.8 (61.5)
<*I*/σ(*I)*>	10.45 (1.95)	10.10 (1.93)	9.09 (2.21)	8.68 (1.97)
**Refinement**				
*R_free_* reflections	2311	3402	2615	2944
No. of atoms (non-H)	42623	41003	40892	41665
Protein	37791	37460	37570	37619
Ligands	426	301	310	341
Solvent	4406	3242	3012	3705
*R*_work_/*R*_free_ (%)	15.8/19.3	17.1/21.1	15.8/21.2	15.4/20.6
Mean ADP^a^ (Å^2^)	34	52	57	47
RMSD from ideal geometry				
Bond lengths (Å)	0.015	0.018	0.016	0.017
Bond angles (^o^)	1.5	1.6	1.7	1.6
Ramachandran statistics (%)				
Favored	96.6	96.3	95.6	96.1
Allowed	3.1	3.4	4.4	3.7
Outliers	0.3	0.3	0.0	0.1
PDB code	5H8I	5H8J	5H8K	5H8L

*Values in parentheses correspond to the highest resolution shell. ^a^ADP, atomic displacement parameter.*

### Structure Determination and Refinement

Initial data of *Mt*CPA were processed to the resolution of 2.65 Å. The online version of *BALBES*, a molecular-replacement pipeline (Long et al., 2008) was used for structure determination. Twenty-one structures with at least 15% identity to the given *Mt*CPA sequence were recruited by *BALBES*. Subsequently, six structures with the best similarity of 39.6% along a 96-residue fragment were used for the structure determination. The following models from the PDB (Berman et al., 2000) were used: 3IVZ, 2W1V, 1F89, 1ERZ, 2VHH and 3AA0. All possible space groups in the orthorhombic system were tested. The best solution was obtained using the model 1ERZ with 574 residues in two monomers. Eight dimers of 1ERZ were found in the asymmetric unit of the *Mt*CPA crystal lattice. The given model and the quality of electron density maps were inspected in *Coot* (Emsley et al., 2010). *PHENIX AutoBuild* (Terwilliger et al., 2008) was used for the model building, starting from the solution obtained from *BALBES*. After rebuilding, the model was taken for the successive steps of manual and automatic refinement with *Coot* and *REFMAC5* (Murshudov et al., 2011). This model was used for the determination of all isomorphous *Mt*CPA and *Mt*CPA-C158S complexes by rigid body refinement. The refinement procedures were following: ligands and solvent molecules were deleted; the atomic displacement parameters were reset to 20 Å^2^ for all atoms and rigid body refinement was used in the first refinement cycle. At the later stages of the structure refinement, TLS parameters (Winn et al., 2001, 2003) were introduced. Restraints for DHMP were generated using *PHENIX eLBOW* (Moriarty et al., 2009) based on the chemical formula, while all the other ligands were refined against standard *CCP4* libraries (Winn et al., 2011). The quality of refined structures was controlled by *R_work_, R_free_* factors (Brunger, 1992) and geometric parameters. *PROCHECK* (Laskowski et al., 1993) and *MolProbity* (Chen et al., 2010) were used for evaluation of the final models. Summary of data collection and refinement statistics are given in **Table 1**.

### Docking

Docking calculations were carried out using Autodock version 4.2.6 (Morris et al., 2009). Non-polar hydrogens were removed from the ligand, and their partial atomic charges were united with the bonded carbon atoms. The docking runs were performed using the Lamarckian genetic algorithm with grid sizes of 60 × 60 × 60 (grid spacing 0.375 Å), yielding 10 docked conformations. During the docking computation, free rotation was allowed around the two C-N aliphatic bonds. Three binding energy terms were taken into account in the docking step: the van der Waals interaction represented as a Lennard-Jones dispersion–repulsion term, the hydrogen bonding term, and the Coulombic electrostatic potential. The resulting 20,000 poses for NCP were analyzed by grouping them into clusters with similar ligand orientations.

### Other Software Used

Molecular illustrations were created with UCSF Chimera (Pettersen et al., 2004). Ramachandran plot was calculated in Rampage (Lovell et al., 2003). Secondary structure was recognized with ProMotif (Hutchinson and Thornton, 1996) within PDBsum server (de Beer et al., 2014). Sequence alignment was performed in CLUSTAL W (Thompson et al., 1994), and edited in BioEdit (Hall, 1999).

## Results and Discussion

### *Mt*CPA Forms Helical Octamers

The polypeptide chain of *Mt*CPA contains 301 amino acids and its calculated molecular weight is 33.8 kDa. *Mt*CPA crystallizes in *P*2_1_2_1_2 space group, with two octamers (chains A-H and I-P) as the asymmetric unit. In each of the structures presented herein, the octamer-flanking monomers (subunits A and H in the first octamer, and I and P in the second) present higher degree of flexibility than the inner subunits of the octamers. This is most probably due to lack of intermolecular contacts, which allows for higher conformational fluctuations. As a result, the electron density maps for the flanking protein subunits (monomers A, H, I, P) were not clear enough to model ligand molecules, and the ligands were placed for 12 out of 16 protein chains (B–G and J–O).

**FIGURE 2:**
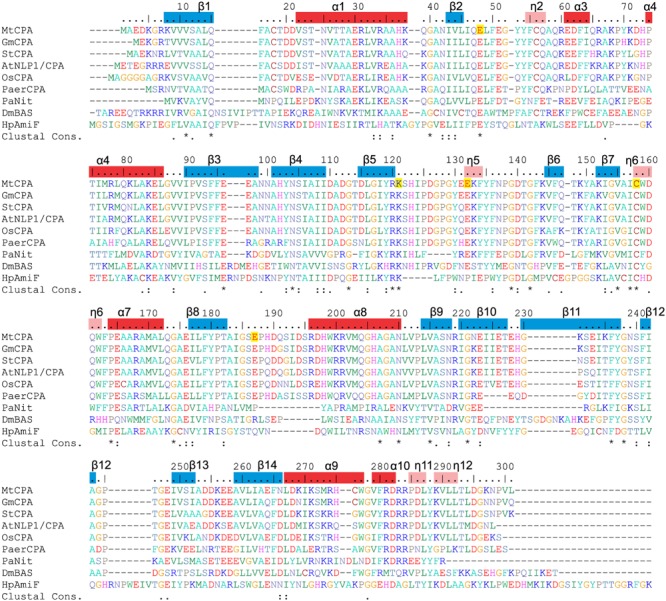
**Sequence alignment of selected nitrilase enzymes.** UniProt accession numbers are given in square brackets, and the values in per cent indicate sequence identity to *Mt*CPA. Six first sequences correspond to CPA enzymes from: *Mt*CPA [G7ITU5]; *Glycine max*, (*Gm*CPA) [I1M4B9] 94%; *Solanum tuberosum*, (*St*CPA) [Q3HVN1] 86%; *Arabidopsis thaliana*, (*At*NLP1/CPA) [B9DGV9] 85%; *Os* Indica group putative protein (*Os*CPA) [A2X5P5] 82%, *Pseudomonas aeruginosa* CPA (*Paer*CPA) [A6UY94] 64%. The other three sequences belong to protein structures described in this manuscript: *Pyrococcus abyssi* Nitrilase (*Pa*Nit) [Q9UYV8] 35%; *Drosophila melanogaster* β-Alanine Synthase without 60 N-terminal residues (*Dm*βAS) [Q9VI04] 31% and *Helicobacter pylori* Formamidase (*Hp*AmiF) [M3MZ63] 27%. Numbering above the sequences and annotation of the secondary structure elements (α helices, red bars; η 3_10_ helices, pink; β strands, blue) corresponds to *Mt*CPA. Residues are color-coded by type, and E48, K121, E132, C158 and E187 in *Mt*CPA sequence are highlighted in yellow.

**FIGURE 3:**
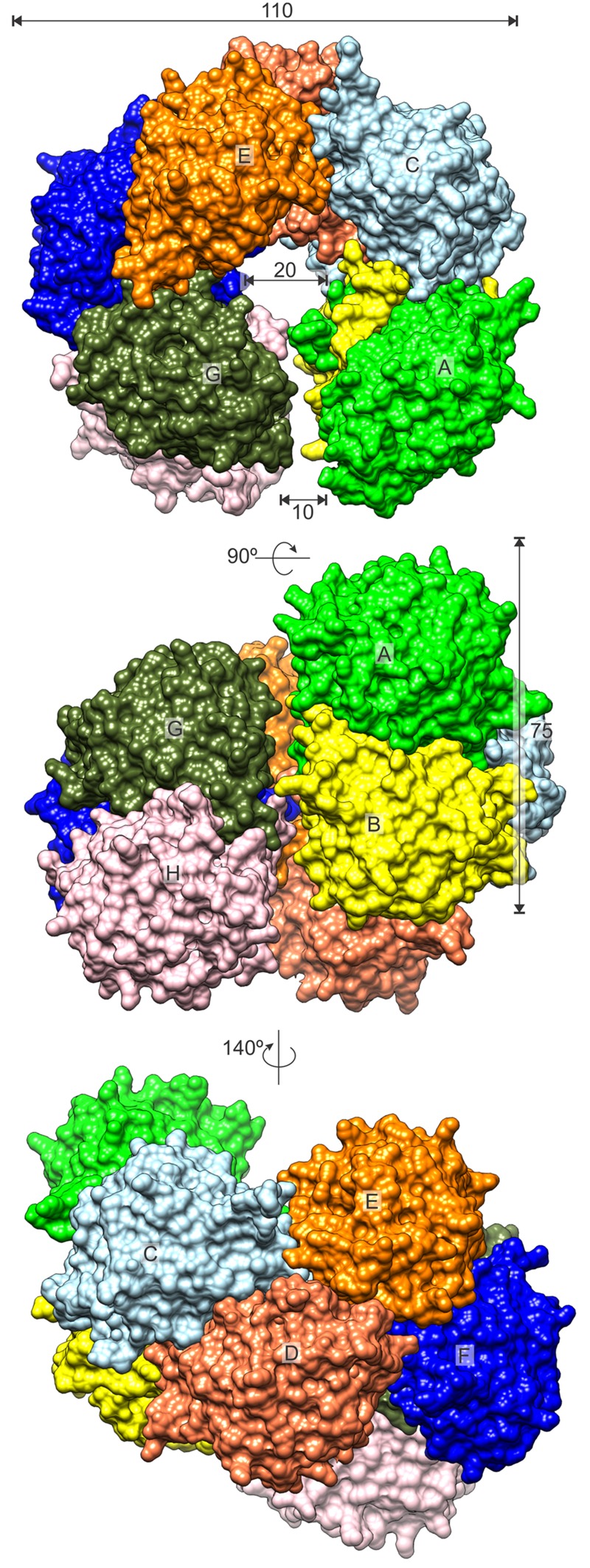
***Mt*CPA octamer.** Surface of each protein subunit (capital letters, A–H) is colored differently. Dimensions are given in Å.

**FIGURE 4:**
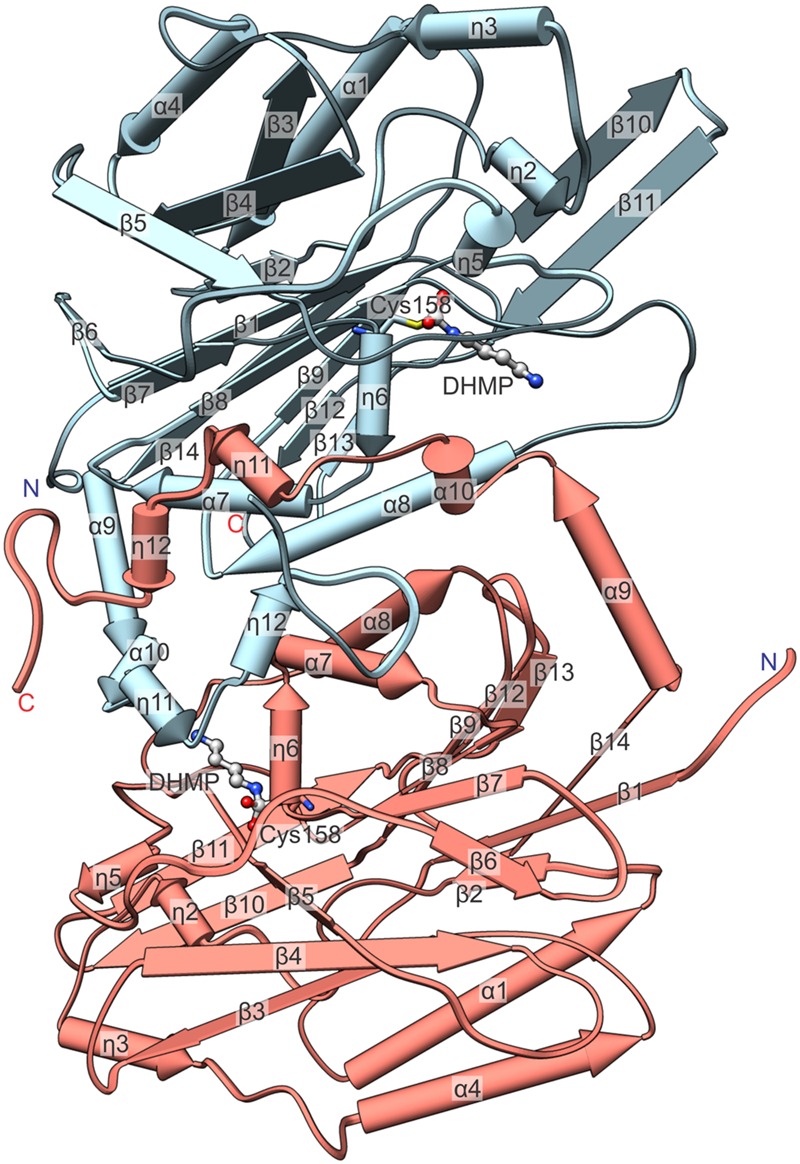
**Detailed view on an isolated *Mt*CPA dimer with annotation of the secondary structure.** Numbering of helices is consecutive, regardless of their type, and η indicate 3_10_ helices. The reaction intermediate, DHMP and the active Cys158 are pictured to visualize the enzymatic reaction venue. Protein chains C (light blue) and D (salmon) are shown.

CPAs show high degree of similarity, especially among plant species (**Figure 2**). Therefore, many conclusions, inferred from the presented structures, may be extrapolated to other CPA enzymes. The octameric quaternary structure in the crystal lattice of *Mt*CPA is in agreement with the assembly reported, based on native gel electrophoresis, for the very close ortholog from *A. thaliana* (85% identity, 92% similarity) (Piotrowski et al., 2003). Based on the crystal structure, *Mt*CPA octamers resemble an incomplete left-handed helical twist with an outer diameter of about 110 Å (**Figure 3**). The helical octamer surrounds a central void of 20 Å diameter. *Mt*CPA octamer can be divided into four symmetric dimers that are shifted by about 30 Å along the long axis of adjacent dimer(s). The gap between the first and the last dimer within the octamer is approximately 10 Å. The dimers present αββααββα topology, and are formed by two monomers of αββα sandwich fold, which is very characteristic for the members of nitrilase superfamily (Nakai et al., 2000; Raczynska et al., 2011). To form a dimer, *Mt*CPA monomers mutually swap their C-terminal fragments from His275 to Leu301 (**Figure 4**) on the inner side of the octamer. The C-terminal swap anchors the monomers to each other in an arm-to-arm manner, sturdily enhancing the monomer-monomer interactions. Furthermore, residues Asp295:Leu301 do not interact with a dimer mate, but instead increase the dimer-dimer interfaces in all but first (A/B and I/J) dimers in each octamer.

The oligomerization state varies among the nitrilases, although they all present a common architecture of αββααββα dimers, with swapped C-terminal fragments analogical to those observed in *Mt*CPA. The length of the swapped fragments varies among proteins with available structures from nitrilase family, from short swap of as little as 10-residue fragment (Kumaran et al., 2003) to longer of about 70 residues (Agarkar et al., 2006). The quaternary assemblies of nitrilases are universally composed of even number of protein subunits. More precisely, the reported functional oligomers vary from dimers (Barglow et al., 2008; Liu et al., 2013), tetramers (Wang et al., 2001), hexamers (Agarkar et al., 2006), octamers, like *Mt*CPA or β*-*alanine synthase from *Drosophila Melanogaster* (*Dm*βAS) (Lundgren et al., 2008), through higher tube-like spiral multimers (Jandhyala et al., 2003; Sewell et al., 2003; Thuku et al., 2007).

In the PDB, one protein of the nitrilase family, *Dm*βAS (Lundgren et al., 2008) (PDB ID: 2VHI), presents helically shaped octamers that resemble those formed by *Mt*CPA. Even though the structures of *Mt*CPA and *Dm*βAS proteins present a similar assembly of the dimers at the first glance, the twist of the *Mt*CPA octamer is more coiled. In contrast, the “uncoiled” *Dm*βAS octamer has a diameter of approx. 120 Å, void of ≈ 30 Å and 65–70 Å pitch (rise along the axis per turn), whereas the corresponding values for *Mt*CPA are: 110, 20, and 45 Å, with the latter value being only theoretical because the pitch of an incomplete helix is virtual. Authors report that *Dm*βAS can freely form higher helical oligomers by attachment of further dimers to the octamer. In contrast, the helix pitch of *Mt*CPA is too small to accommodate another dimer (≈ 75 Å along the long axis), and the addition of further dimers would either create severe steric clashes or force significant structural rearrangements (uncoiling) of the octamer. Consistently, the flanking dimers of the *Mt*CPA octamer are separated by only a 10 Å gap, much narrower than in the *Dm*βAS octamer. In *Dm*βAS, the gap between the first and last dimer is almost 40 Å, which is sufficient to accommodate additional dimer, necessary for the formation of the theoretically infinite helical assembly.

### Complexes with: The Reaction Intermediate, CAD and the Structure of *Mt*CPA-C158S Mutant and Its Complex with PUT

**FIGURE 5:**
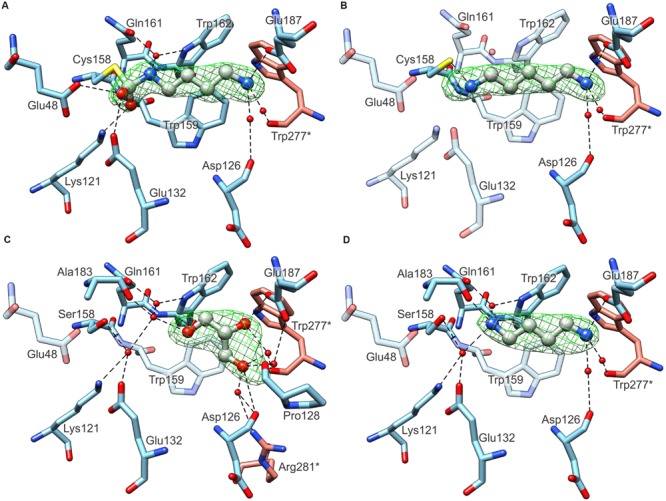
**Ligand binding by *Mt*CPA **(A,B)** and *Mt*CPA C158S mutant **(C,D)**.** In each panel protein subunits C (blue C atoms) and D (salmon) are shown whereas the ligands: DHMP in **(A)**; CAD **(B)**; PUT **(C)** and GOL **(D)** are in gray. Green meshes visualize omit electron density maps contoured at 5 σ level around ligands. Dashed black lines indicate hydrogen bonds. Semitransparent residues in **(B–D)** do not interact with ligands, and are depicted for comparison only.

The structures of wild-type *Mt*CPA, crystallized in the presence of PUT and CAD in the solution, were determined at 1.97 and 2.19 Å resolution, respectively. Inspection of the electron density maps, representing the structure of *Mt*CPA cocrystallized with PUT, brought unexpected, but very informative results. Instead of linear PUT, the reaction intermediate, DHMP, was observed in the active site (**Figure 5A**). More interestingly, DHMP was covalently bound to the catalytic Cys158. There are two conceivable scenarios of the DHMP adduct formation during crystallization. It is possible that PUT reacts with carbon dioxide (or carbonic acid) in solution, outside the catalytic site of the enzyme, for it is known that primary amines react with CO_2_ in aqueous solution (Penny and Ritter, 1983; McCann et al., 2009). The resulting *N*-carbamicputrescine (with carboxylic COO^-^ group attached to one of the PUT amines) may enter the active site and, similarly to the NCP, most likely is susceptible to the nucleophilic attack of the Cys158. The DHMP covalent adduct is stabilized by placement of one of its oxygen atoms within the oxyanion hole, created by Nζ of Lys121 and the backbone N of Trp159 (see Active Site and the Reaction Mechanism). The other scenario of events, which might lead to the covalent enzyme-DHMP adduct in the crystallization drop, involves an initial reaction of carbon dioxide (or carbonic acid) with the active Cys158. To our judgment, however, this situation is much less likely for two reasons. Firstly, the covalent C-N bond in that case would need to result from a nucleophilic attack of N atom of PUT on the S-bound carboxyl C, which is very questionable. Secondly, if the second scenario was true, one would expect a similar state in the structure of *Mt*CPA cocrystallized with CAD, because nothing should prevent the Cys158 carboxylation in the first place. In that instance, there would be not enough room to bind entire CAD molecule. This is not the case, and we do indeed observe CAD in the active site (**Figure 5B**). It is, of course, possible that CAD molecules in solution also form CO_2_ adducts, but due to steric clashes (see Substrate Specificity), they cannot be recruited to the catalytic site, meanwhile non-modified CAD molecules can. CAD in *Mt*CPA complex reaches deeper than PUT into the catalytic pocket, however, due to presence of sulfhydryl group of Cys158 it bends toward Glu132.

The crystal structure of wild-type *Mt*CPA without addition of any ligands was determined at 2.55 Å resolution (data not shown). Unfortunately, the electron density maps for the content of the catalytic site were unclear, and represented most probably disordered ingredients of the crystallization solution, GOL, and partially oxidized Cys158. Combined with the rather poor data resolution, we were not able to unambiguously interpret the content of the active site of “unliganded” wild-type *Mt*CPA, and we do not report this structure herein. Instead, we decided to construct the *Mt*CPA-C158S mutant. Substitution of sulfhydryl with hydroxyl group preserved binding properties of residue 158 but strongly diminished its reactivity. The structure of unliganded *Mt*CPA-C158S, determined at 2.39 Å resolution, presented significantly lower noise in the electron density maps around the active site when compared to the unliganded wild-type *Mt*CPA. This improvement allowed to identify the ligand bound inside the active site as GOL (**Figure 5C**). However, GOL does not penetrate deep inside the pocket and presents no direct interactions with either of the residues from catalytic triad. Instead, it is hydrogen-bonded with the side chain oxygen of Glu187.

The crystal structure of *Mt*CPA-C158S mutant cocrystallized with PUT clearly shows the ligand without any modifications (**Figure 5D**). In this case, the absence of nucleophilic sulfur prevents from the formation of covalent bond between DHMP and residue 158. In fact, PUT in *Mt*CPA-C158S complex does not interact directly with the residue 158, but creates water-mediated hydrogen bond with the hydroxyl group of Ser158 instead.

### Active Site and the Reaction Mechanism

**FIGURE 6:**
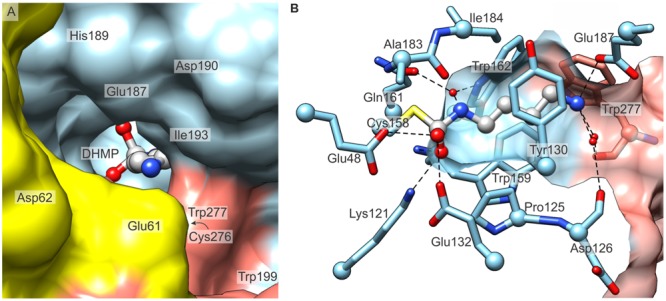
**The active site of *Mt*CPA. (A)** View along the entrance to the cavity which contains the active site, and the reaction intermediate, DHMP inside. Protein subunits B (yellow), C (light blue) and D (salmon) are shown. **(B)** Detailed mode of interaction of *Mt*CPA with DHMP. Dashed lines indicate hydrogen-bonded atoms. Hydrogen bonds mediated by more than one water molecule are omitted for clarity. Cα atoms of interacting residues are shown as balls. The protein surface (semitransparent, C and D) is clipped to show the maximum vista over the binding cavity.

Each *Mt*CPA monomer contains a cavity, which is accessible from the outer surface of *Mt*CPA octamer and, in its depth, comprises the active site. In order to present the active sites in a clear manner, we divide the *Mt*CPA cavities to four parts, each buried deeper in the protein core: (i) the entrance preceding the genuine active site; (ii) the tail amine-binding region, which binds the non-reacting amine group of PA; (iii) the non-polar section, interacting with the ligand aliphatic fragment; and (iv) the catalytic triad, which performs the reaction. The cavities are in a very close proximity to the neighboring protein subunits, with the exception of the outer monomers A, H, I, and P that only have a single neighbor. The entrances to the funnel-like catalytic cavities are actually shaped not only by the amino acids from the protein subunit to which a particular site belongs, but are also fenced by protein chains of its immediate neighbor(s) (**Figure 6**). Moreover, each dimer-mate subunit surrounds a larger fragment of the active site entrance than the protein chain from the neighboring dimer.

Glu187, Asp190, together with several carbonyl oxygens of Trp277^∗^ (asterisk indicates a residue that belongs to dimer-mate subunit), Cys276^∗^, Asp126 and Pro128 are responsible for the negative potential localized around the active site (**Figure 6A**). Most probably, they attract the positively charged NCP, when it penetrates its way through the cavity. Walls of the middle section of the active site are composed of the side chains of: Tyr130, Tyr54, Pro125, Trp159, Trp162, Ile184, Ala183, and by Trp277^∗^ from the swapped C-terminal fragment (**Figure 6B**). In this manner, *Mt*CPA provides the hydrophobic milieu of the central section of the active pocket. This fragment is, therefore, perfectly adapted to bind the aliphatic fragment of the substrate, NCP by hydrophobic interactions.

As in other nitrilases, the substrate hydrolysis is carried out by the catalytic triad, composed in *Mt*CPA of Glu48 (proton acceptor), Lys121 (proton donor) and Cys158 (nucleophile). These three residues are placed deep inside the narrow part of the active site. The nucleophilic Cys, as in any other nitrilase-like protein, is positioned at the apex of the nucleophile elbow (Kumaran et al., 2003), formed in *Mt*CPA by β7-turn-η6 motif, and its tightness enhances the nucleophilic character of Cys158 by enforcing energetically unfavorable conformation. The nucleophilic character of the active Cys158 is additionally fortified by two glutamates, Glu48 and Glu132, which attract the sulfhydryl proton. Both glutamates, i.e., Glu48, as the member of the catalytic triad, but also Glu132 are highly conserved among nitrilase superfamily. Most probably, this pair attracts the NH_2_ group of the NCP, therefore, properly orients the carbamoyl carbon of the substrate for the nucleophilic attack by Cys158. Moreover, Glu48 and Glu132 affect the closest environment of Lys121 and preserve its positive charge, crucial for the hydrolysis.

**FIGURE 7:**
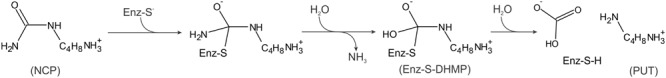
**A scheme of enzymatic hydrolysis of NCP to PUT.** Enz-S-DHMP indicates the covalent complex with *N*-(dihydroxymethyl)putrescine, observed in the *Mt*CPA/DHMP structure.

The hydrolysis of NCP by *Mt*CPA most likely follows a mechanism similar at first stages to the proposed earlier for *Helicobacter pylori* formamidase (*Hp*AmiF) (Hung et al., 2007), but after creation of the oxyanion geminal diol intermediate (see below), it carries on in its own way. Briefly, activated Sγ of Cys158 performs nucleophilic attack on the carbamoyl C atom of the substrate, creating the covalent, tetrahedral (geometry concerns the initially planar C atom, which ultimately leaves as HCO_3_) diaminothioalcoholate intermediate (**Figure 7**). The generated oxyanion is stabilized by the oxyanion hole, formed by Nζ of Lys121 and backbone N of Trp159, similarly to the case reported for *Pyrococcus abyssi* nitrilase (*Pa*Nit) (Raczynska et al., 2011). Subsequently, the amino group of the intermediate accepts a proton, donated by Glu48, and leaves as the first by-product, ammonia, resulting in the planar *S*-acyl intermediate-enzyme complex, which is hydrated to yield the tetrahedral oxyanionic geminal diol (present in the crystal structure of *Mt*CPA/DHMP complex). Then, most probably, the water molecule tethered to O𝜀 of Gln161 and N𝜀 of Trp162 is added to the intermediate, and, in an apparently concerted manner, C-S and C-N bonds are cleaved, which results in the release of PUT and the second by-product, HCO_3_^-^.

### Substrate Specificity

Enzymatic assays on CPA enzymes from *At* (Piotrowski et al., 2003) and *Pseudomonas aeruginosa* (*Paer*CPA, Nakada and Itoh, 2003) show that CPAs are highly active only against NCP. Plant *At*CPA showed no activity toward substrates of other nitrilase-like enzymes, e.g., amino acids, *N*-Carbamoyl-β-alanine, agmatine. Also, the bacterial *Paer*CPA very poorly hydrolyzed both longer and shorter substrates in comparison to NCP: *N-*carbamoylcadaverine and *N-*carbamoyldiaminopropane, respectively.

**FIGURE 8:**
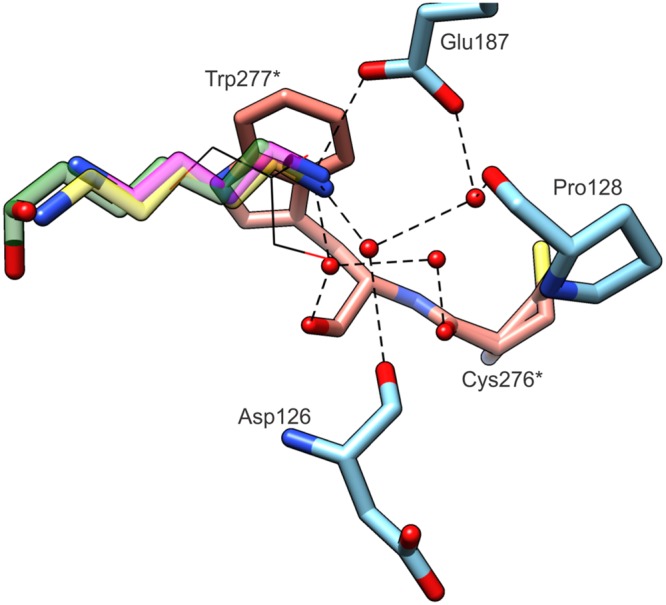
**Substrate specificity of *Mt*CPA.** The ligands (semitransparent) inside the cavity originate from *Mt*CPA/DHMP complex (green); *Mt*CPA/CAD (yellow); *Mt*CPA-C158S/PUT (magenta). GOL molecule (black wires) is superposed from *Mt*CPA-C158S unliganded structure. Interacting amino acid residues and hydrogen-bonding network (dashed black lines) are shown for *Mt*CPA/DHMP complex only, but their locations are preserved in *Mt*CPA/CAD and *Mt*CPA-C158S/PUT complexes as well. Asterisks (^∗^) indicate amino acid residues that belong to dimer-mate subunit. Interactions mediated by more than two water molecules (red balls) are omitted for clarity.

We addressed the question of structural features that are responsible for such narrow substrate specificity of CPA enzymes. The comparison of the product (PUT), its longer analog (CAD) and the intermediate (DHMP) bound within the active site of *Mt*CPA highlights key elements of the active site architecture, which determine the enzyme preference for NCP (**Figure 8**). All bound amines, CAD, PUT and DHMP, present the same hydrogen bond between terminal amine group and the side chain oxygen of Glu187. Additionally, these amino groups create two water-mediated hydrogen bonds with carbonyl oxygens of Asp126, Trp277^∗^, and mediated by two waters hydrogen bonds with Pro128 and Cys276^∗^. Glu187, conserved in CPA enzymes, prevents from substrate mismatch, and stabilizes substrate position within the active site. Moreover, the position of negatively charged Glu187 at the active site entrance determines both the charge and length of the bound substrate. Longer or shorter molecules than NCP, would not be able to create a hydrogen bond with the oxygen of Glu187, and/or would collide with other parts of the active site.

The pocket presents very narrow and highly hydrophobic middle section, which excludes bulky substrates form binding. It also limits the spectrum of possible substrates to those which have an unbranched aliphatic fragment of four carbon atoms. From the *Mt*CPA/CAD complex, we can assume that longer substrates than NCP, such as *N*-carbamoylcadaverine, would not obtain stable conformation inside the active site. Although CAD is able to interact *via* hydrogen bond with Glu187 at the entrance of the pocket, similarly to PUT and DHMP, deep inside the cleft CAD passes by the Sγ of Cys158 and bends toward the pair of glutamates. Thus, this would not leave enough space for the carbamoyl moiety of such longer substrate. In other words, the proper orientation of *N*-carbamoylcadaverine, with the preservation of the hydrogen bond with the Glu187 side chain is impossible due to steric clashes either with the catalytic triad or with the residues in the middle section of the pocket.

The commercial availability of NCP is extremely limited, therefore, during our studies, we attempted to obtain complexes with other ligands, such as urea, sarcosine, L-lysine, L-arginine, which would mimic the substrate. Unfortunately, their complexes with neither wild-type *Mt*CPA nor its C158S mutant allowed to unambiguously identify any other ligand of interest within the active site, except for those presented in this paper.

### Proposed Secondary Binding Site

As it was shown by Piotrowski et al. (2003), the hydrolysis of NCP by CPA from *A. thaliana* does not follow the Michaelis–Menten kinetics. Instead, in the excess of substrate, an accelerating mechanism is triggered. Since PAs are linked with the plants response to stress factors, this phenomenon may actually be significant *in vivo*. The positive allosteric regulation was found also in other nitrilase-like proteins, β-alanine synthases (Tamaki et al., 1987; Matthews et al., 1992; Sakamoto et al., 2001; Lundgren et al., 2008). In these enzymes, the mechanism was attributed to the change of oligomerization caused by increasing substrate concentration, rather than to the existence of an allosteric regulatory site (Matthews et al., 1992). However, in *Mt*CPA, assuming that it also follows the Hill kinetics, due to disabled formation of higher oligomers, a different mechanism may control the enzyme behavior.

**FIGURE 9:**
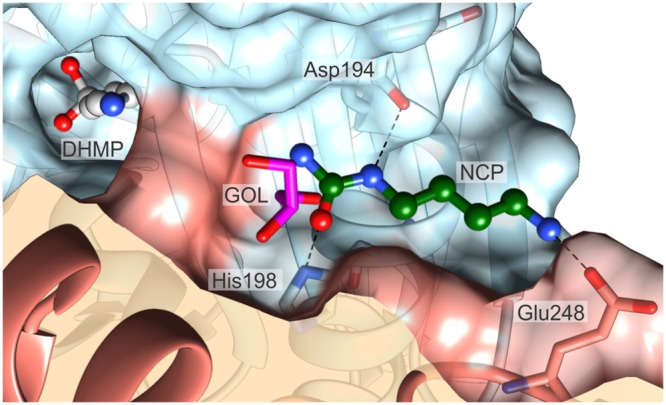
**The proposed alternative substrate-binding site.** The substrate (ball-and-sticks, green C atoms) was docked at the location where a GOL molecule (magenta) is present universally in all presented crystal structures. Note that the carbamoyl moiety of the substrate partially overlaps with the GOL position. The reaction intermediate, DHMP (ball-and-sticks, gray), bound in the catalytic center is shown to visualize the structural proximity of the two sites. Dashed black lines indicate possible hydrogen bonds with Asp194, His198 and Glu248. The surfaces of the two dimer-forming protein subunits are color-coded red and blue.

The architecture of close neighborhood of the catalytic site shows a solvent/cytoplasm accessible niche near the substrate binding pocket. In every structure presented herein, this niche is occupied by a GOL molecule. Considering the fact that in the *Mt*CPA-C158S mutant structure, GOL enters the active site, and mimics the substrate or product, the niche-bound GOL quite likely might represent the secondary binding site. In that case, in higher concentration of NCP, this site might be occupied by the substrate. Docking simulation seems to support this hypothesis. In fact, the carbamoyl moiety of the docked substrate, NCP, significantly overlaps with the GOL-binding site from the presented here complexes (**Figure 9**). The substrate is situated in the niche with the carbamoyl moiety aiming at the entrance to the active site. In the predicted alternative binding site, NCP, would form three hydrogen bonds with Asp194, His198 and Glu248^∗^. The calculated binding energy of the secondary binding site is -3.6 kcal mol^-1^, whereas the corresponding value for the genuine catalytic cavity equals -5.8 kcal mol^-1^. It is, therefore, suggestive that the substrate affinity is significantly lower in the alternative binding site, nonetheless, still within a reasonable range.

At this point, however, the mechanism of allosteric regulation remains unrevealed, for it appears that binding of the second NCP molecule, should neither directly facilitate binding of the substrate within the active site, nor it should impact the catalytic triad. It is possible that the alternative NCP binding location represents a standby spot for substrate molecule. Even though it would not be an allosteric regulation of the enzyme activity *per se*, it would mitigate the delay needed for the substrate diffusion into the active site. By far, it is merely a speculation, and many additional experiments are required to validate this hypothesis, however, it is worth to mention that a standby substrate position was reported, e.g., for tyrosine phosphatase from *Selenomonas ruminantium* (Puhl et al., 2007), unrelated to CPA proteins.

### Conclusions, Physiological Significance and Future Outlook

This study was focused on the structural characterization of CPA from the model legume plant, *M. truncatula*. The octameric *Mt*CPA enzyme is assembled into an incomplete helical twist, made by four tight dimers with swapped C-termini. This dimeric αββααββα organization is highly conserved among nitrilase-like enzymes, however, the analogical octameric assembly was observed only in *Dm*βAS (Lundgren et al., 2008).

The octameric structure of plant CPAs, reported previously for *A. thaliana* (Piotrowski et al., 2003) and herein for *M. truncatula* brings up a fundamental question of physiological benefit from forming high oligomers. The role of PUT in response to abiotic stresses (mentioned in the Introduction), such as high salinity, drought and/or thermal extrema, could provide a conceivable answer. Assembling into higher oligomers undoubtedly increases enzyme’s tolerance to adverse conditions, for a smaller portion of the protein surface is exposed toward the cytoplasm and, therefore, is less susceptible to changing environment. A similar rationale was anticipated to explain oligomerization of other abiotic-stress-related plant proteins, which form ring-like decamers, δ^1^-pyrroline-5-carboxylate reductases (Forlani et al., 2015).

*N*-carbamoylputrescine amidohydrolases are enzymes highly specific towards NCP (Piotrowski et al., 2003), and the architecture of the *Mt*CPA catalytic cavity, formed as a deep pocket with broadened entrance, explains this phenomenon. The entrance and its middle section is built with a contribution of the residues from neighboring monomers Cys276^∗^ and Trp277^∗^. Two characteristic features influence the substrate specificity of *Mt*CPA: the narrow and hydrophobic channel in the middle section of the catalytic cavity (Tyr130, Tyr54, Pro125, Trp159, Trp162, Ile184, Ala183 and Trp277^∗^ from dimer mate) and Glu187, placed at the entrance, the key residue responsible for the proper substrate orientation inside the active site. The catalytic triad at the bottom of the cavity is composed of Glu48, Lys121 and Cys158. Glu48, the proton acceptor, in the cooperation with Glu132 enhances the nucleophilic character of Cys158 and maintains the proper charge of Lys121, the proton donor. Lys121, together with backbone N of Trp159 forms the oxyanion hole, which stabilizes the intermediate state during NCP hydrolysis. Cys158, placed at the apex of the nucleophile elbow, formed in *Mt*CPA by β7-turn-η6 motif, is responsible for the nucleophilic attack on the substrate, and creation of the covalent intermediate-enzyme complex. The catalytic water molecule, which performs subsequent nucleophilic attack on the intermediate, is stored within the active site in a small niche, near the catalytic triad. This water is hydrogen-bonded to the side chains of Gln161 and Trp162. The reaction leads to the breakage of two covalent bonds of the substrate, which ends with the release of PUT, ammonia and carbon dioxide. Presented here indication of the key residues, responsible for specificity and activity of *Mt*CPA, sheds new light on CPA enzymes overall. Any future in-depth physiological study of CPAs can be more rational, as the research can benefit, or even directly arise from the structural knowledge presented herein.

The crystallographic analysis of the complexes of *Mt*CPA and its C158S mutant confirmed high affinity of their active sites for ligands, which were either absorbed from crystallization solution (GOL), or intentionally added to obtain *Mt*CPA complexes (PUT and CAD). Interestingly, in the complex of the wild-type *Mt*CPA, obtained by the cocrystallization with PUT, the reaction intermediate, DHMP, was covalently bound to the active Cys158, inside the active site instead of PUT. DHMP adduct was most probably formed as the result of the reaction of the amine group of PUT with carbon dioxide, absorbed by the crystallization drop, followed by nucleophilic attack of the active Cys158. This showed that the oxyanion hole, next to the *Mt*CPA catalytic triad, is able to stabilize the otherwise labile geminal diol. It would be very interesting to see whether a similar scenario could be relevant *in planta*, that is, if CO_2_ to a significant extent reacts with PUT in the cytoplasm and (as carbamic acid) enters the catalytic cavity of *Mt*CPA. If so, the enzyme activity would be modulated by CO_2_ concentration, which would give another link between PAs and plant stress response.

Several members of nitrilase superfamily have been previously reported to exert enzymatic mechanism suggesting cooperative binding. Due to lack of a candidate secondary substrate-binding position, for most of them this behavior was associated with changes of the oligomerization state. *Mt*CPA, however, is not able to change the oligomerization state, as its octamers are formed in a very coiled manner. We examined *Mt*CPA structure to indicate a secondary substrate-binding site. The analysis led to designation of the conserved (in *Mt*CPA structures) GOL binding location as the potential alternative substrate binding site. This hypothesis was supported by docking experiment, which indicated that the substrate may indeed bind also in a niche, with carbamoyl moiety of the docked NCP partially overlapping the GOL binding site. This venue is very close to the entrance of the active site, but separated from the catalytic triad. However, with present data we are not able to unambiguously determine the activity-accelerating mechanism linked to the alternative binding site. We may suppose that *Mt*CPA might not be allosterically regulated *per se*, but rather own a site, where the substrate can standby, prior to entering the catalytic site. In that case, the turnover rate of the enzyme would be increased, as the delay needed for the substrate to diffuse in the cavity would be alleviated if it was bound right next to the entrance. This hypothesis, however, needs to be verified using parallel functional and structural approaches, nonetheless, it sketches a perspective for future studies of CPA enzymes. From the physiological perspective, accelerating mechanism is very beneficial, as it triggers increased enzyme activity when it is most needed, bypassing regulatory mechanisms of transcription and translation. During abiotic and biotic stresses as well as in the course of nodulation, when the demand for PAs is aggravated, plant can produce sufficient amount of PUT without engaging the protein synthesis machinery to deliver more molecules of CPA, but instead simply providing more NCP.

### Accession Numbers

Coordinates and structure factors of the related structures were deposited in the PDB: *Mt*CPA/DHMP complex, 5H8I; *Mt*CPA/CAD, 5H8J; *Mt*CPA-C158S mutant, 5H8K; *Mt*CPA-C158S/PUT, 5H8L.

## Author Contributions

BS produced the protein and solved the crystal structures. BS, MR, and ZD analyzed the data. MM performed docking calculations. MR and BS wrote the manuscript.

## Conflict of Interest Statement

The authors declare that the research was conducted in the absence of any commercial or financial relationships that could be construed as a potential conflict of interest.

## Abbreviations

ADC: arginine decarboxylase
AIH: agmatine iminohydrolase
*At*: *Arabidopsis thaliana*
CAD: cadaverine
cDNA: complementary DNA
CPA: *N*-carbamoylputrescine amidohydrolase
*Dm*βAS: *Drosophila Melanogaster* β-alanine synthase
DHMP: *N*-(dihydroxymethyl)putrescine
GOL: glycerol
*Gm*: *Glycine max*
*Hp*AmiF: *Helicobacter pylori* formamidase
IPTG: isopropyl-β-D-thiogalactopyranoside
*Mt*: *Medicago truncatula*
NCP: *N*-carbamoylputrescine
ODC: ornithine decarboxylase
*Os*: *Oryza sativa*
PA: polyamine
*Paer*: *Pseudomonas aeruginosa*
*Pa*Nit: Nitrilase from *Pyrococcus abyssi*
PCR: polymerase chain reaction
PDB: Protein Data Bank
PUT: putrescine
SPD: spermidine
SPM: spermine
*St*: *Solanum tuberosum*;
TCEP: tris(2-carboxyethyl)phosphine
TEV: Tobacco Etch Virus
